# Risk factor analysis of growing unruptured intracranial aneurysms: A single center cohort study

**DOI:** 10.1016/j.bas.2026.105972

**Published:** 2026-02-08

**Authors:** Francis J. Kissling, Johannes Goldberg, Bettina L. Serrallach, Michael Murek, Tomas Dobrocky, Eike I. Piechowiak, Jan Gralla, Andreas Raabe, David Bervini

**Affiliations:** a University of Bern, Bern, Switzerland; b Department of Neurosurgery, Inselspital, Bern University Hospital, Bern, Switzerland; c Department of Diagnostic and Interventional Neuroradiology, Inselspital, Bern University Hospital, Bern, Switzerland

**Keywords:** Aneurysm growth, Intracranial aneurysm, Survival analysis, UIA, Risk factors

## Abstract

**Introduction:**

Intradural unruptured intracranial aneurysms (UIA) can rupture and lead to subarachnoid hemorrhage. Previous studies suggest aneurysm growth being a strong risk factor for rupture. The natural history and the individual risk of growth of aneurysms remain controversial.

**Research question:**

To analyze the time to growth and identify risk factors associated with growth of UIAs.

**Materials and methods:**

Prospectively collected data of 588 patients with a total of 858 UIAs followed up by imaging were analyzed. Patients and aneurysms were categorized in a growing or a stable cohort. Logistic and survival analyses were used to assess potential factors associated with UIA growth.

**Results:**

During a median follow-up of 3.4 years (IQR 1.3 – 6.6 years), 112 (13.1%) out of the 858 UIAs showed an increase in size or a change of morphology. Posterior circulation UIAs (OR 2.01 (1.15 – 3.52), *p* = 0.01), aneurysm size at diagnosis (OR 1.10 (1.04 – 1.16) per mm, *p* = 0.002) and arterial hypertension (OR 1.60 (1.03 – 2.49), *p* = 0.04) were significantly associated with growth. Survival analyses confirmed a time-dependent association with growth for posterior circulation UIAs (HR 3.9 (1.74 – 8.75), *p* = 0.002) and aneurysm size (HR 1.22 (1.13 – 1.31), *p* < 0.0001).

**Discussion and conclusion:**

UIA location in the posterior circulation, a larger size at diagnosis and the presence of arterial hypertension are significant risk factors associated with UIA growth.

## Highlights

• Logistic and survival analysis of n = 588 patients with 858 aneurysms.
• Posterior circulation aneurysms at a higher risk of growth.
• A larger aneurysm size at diagnosis increases the risk of growth.
• Presence of arterial hypertension associated with aneurysmal growth.

## List of abbreviations

(a)SAH: (aneurysmal) subarachnoid hemorrhage
ACA: anterior cerebral artery
ADPKD: autosomal dominant polycystic kidney disease
AICA: anterior inferior cerebellar artery
BA: basilar artery
CI: confidence interval
CT/CTA: computed tomography/angiography
DSA: digital subtraction angiography
FU: follow-up
HR: hazard ratio
ICA: internal carotid artery
IQR: interquartile range
MRI/MRA: magnetic resonance imaging/angiography
MCA: medial cerebral artery
NF: neurofibromatosis
n: number
n/a: not available
OR: odds ratio
PCA: posterior cerebral artery
PcomA: posterior communicating artery
PICA: posterior inferior cerebellar artery
SCA: superior cerebellar artery
SAH: subarachnoid hemorrhage
SD: standard deviation
UIA: unruptured intracranial aneurysm
VA: vertebral artery

## 1 Introduction

Unruptured intracranial aneurysms (UIA) are found in approximately 3.2 percent of the population (Vlak et al., 2011). While most aneurysms remain asymptomatic and are either not discovered at all or diagnosed incidentally, some intradural intracranial aneurysms do rupture and lead to subarachnoid hemorrhage (SAH) which is associated with high morbidity and mortality (Goldberg et al., 2018, 2023; Schatlo et al., 2021). To prevent aneurysmal rupture, UIAs can be occluded by either microsurgical or endovascular techniques. However, these methods carry a risk of neurological complications (Alshekhlee et al., 2010; Kotowski et al., 2013; Wiebers, 2003). Therefore, treatment-associated risks need to be balanced against the natural risk of rupture in a patient-specific setup. If treatment-associated complication risks are considered to exceed cumulative rupture risks in an individual patient, conservative treatment with repeated follow-up imaging is recommended (Thompson et al., 2015). An increase in UIA size and/or change in morphology has shown to be a strong risk factor for the rupture of UIAs in different studies (Mehan et al., 2014; Chmayssani et al., 2011; Villablanca et al., 2013; Brinjikji et al., 2016).

Although the natural history of UIAs has already been subject to research, it remains controversial. So far, different patient- and aneurysm-related risk factors for aneurysmal growth have been discussed in the literature, such as UIA size, smoking, multiplicity, female sex, location and shape of the aneurysm (Brinjikji et al., 2016; Backes et al., 2016; Jin et al., 2019). In recent years, the importance of hemodynamic factors as well as resulting vessel wall inflammation and their role in aneurysmal formation, growth and rupture has been brought into focus as well (Cebral et al., 2017; Leemans et al., 2019; Frösen et al., 2019; Wang et al., 2019). Due to the limited numbers of patients in many studies and heterogenous follow-up patterns, results are inconsistent throughout the literature.

The aim of this study was to strengthen the existing evidence on reliable risk factors and to analyze the rate and the timing of growth of UIAs in a large single centre cohort.

## 2 Methods

### 2.1 Study design and population

Consecutive patients diagnosed with one or more UIAs admitted to the University Hospital of Bern, Switzerland are discussed in a weekly neurovascular aneurysm board meeting of interventional neuroradiologists and neurosurgeons. Treatment recommendations are recorded prospectively. Records from the first protocol in November 2013 until December 2021 were analyzed for patients with repeated follow-up imaging. A total of 748 consecutive patients were initially recruited for the analysis.

#### 2.1.1 Exclusion criteria

**Fig. 1:**
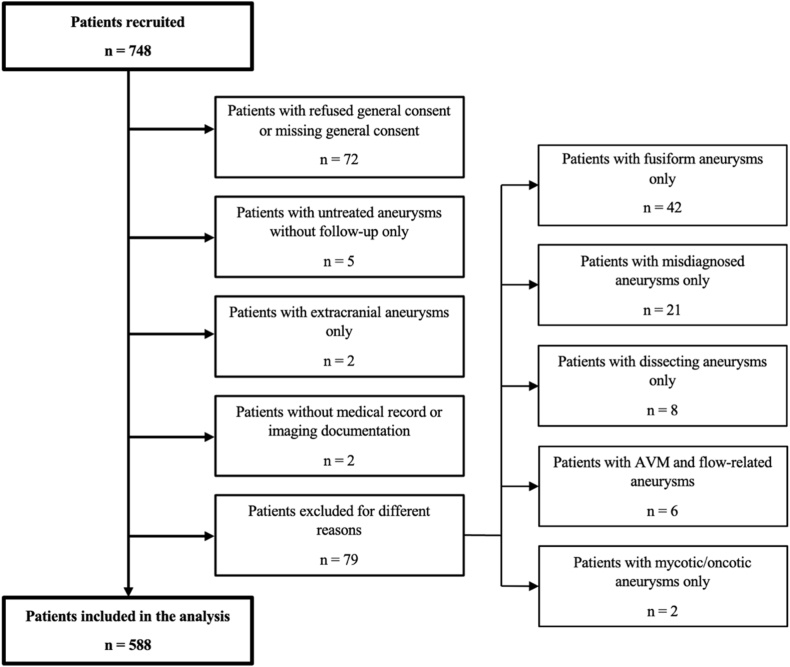
Flow chart of the patient selection process.

Patients with refused or missing general consent, without follow-up imaging, with extradural aneurysms only, fusiform aneurysms only, pseudoaneurysms following an arterial dissection only, arteriovenous malformation (AVM) and flow-related aneurysms and oncotic or mycotic aneurysms only were excluded from our study. Two patients with missing medical records were excluded as well. Fig. 1 illustrates the patient selection process.

### 2.2 Data collection

Patient- and aneurysm-related variables were prospectively documented and retrospectively inserted in a computerized online database (REDCap powered by Vanderbilt University, Nashville Tennessee, USA; electronic data capture tools hosted by the CTU at the University of Berne, Version 15.0.11) (Harris et al., 2009, 2019).

#### 2.2.1 Patient-related variables

Patient-related variables retrieved from electronic patient records included sex, age at time of aneurysm diagnosis, smoking status, history and treatment of arterial hypertension, history of aspirin medication (positive if intake during >50% of the follow-up time), family history of UIA or SAH (positive if ≥ 2 first-degree relatives with diagnosis of UIA and/or SAH), history of autosomal dominant polycystic kidney disease (ADPKD) and other connective tissue disorder as well as personal history of SAH.

#### 2.2.2 Aneurysm-related variables

Aneurysm- and imaging-related data was collected from the picture archiving and communication system PACS Sectra IDS7 (Sectra AB, Linköping, Sweden): aneurysm location (anterior vs. posterior circulation), imaging modality (computed tomography angiography CTA, magnetic resonance angiography MRA or digital subtraction angiography DSA), maximum size (defined as the maximum dome diameter in any aneurysm projection) and aneurysm morphology compared to the previous image (stable vs. growing). Aneurysm size was retrieved from the radiological reports. Growth was defined as any reported size and/or morphological change, without a standardized size change threshold. In case of contradictory reports, images were reviewed by two authors (F.J.K. and B.L.S.). Aneurysms without definite change of size or morphology to be measured or seen, were classified as stable.

#### 2.2.3 Cohorts

Patients and aneurysms were categorized in a stable or a growing cohort. In the per-patient analysis, patients with at least one growing aneurysm were considered in the growing cohort, even if they had other stable aneurysms.

#### 2.2.4 Regular interval subgroup

As imaging intervals varied substantially, a subgroup with regular follow-up of a maximum of 2 years interval between imaging studies was defined.

#### 2.2.5 Incidence rate analysis

Incidence counts and rates at different follow-up phases were calculated based on the time to growth or the total follow-up time in cases of non-growing aneurysms. The “growing UIA count” was defined as the number of occurrences of growth during a certain time period, the “total UIA count” as the total number of aneurysms with a censoring point during that follow-up time period. Censoring was defined as the first occurrence of growth (event) *or* as the last imaging study in non-growing aneurysms. The number of “UIA at risk” was defined by the number of aneurysms included at the beginning of each time span. Aneurysm-years at risk were calculated for each time span separately as well as for the total cohort. Individual incidence rates for each time period were calculated as follows: $incidence\;rate=\frac{count\;of\;new\;occurrences\;of\;growth}{aneurysm\;years\;at\;risk}$.

### 2.3 Imaging protocols

Most aneurysms were first diagnosed incidentally on CTA or MRA scans performed for other indications, sometimes confirmed by DSA. Neurovascular board members decided on follow-up imaging modality and interval. During the second half of the screened time period, institutional guidelines and follow-up recommendations were introduced.

### 2.4 Statistical analysis

Statistical analyses were conducted in a per-patient and/or a per-aneurysm based manner. Descriptive statistics included means with standard deviation (SD), medians with lower and upper quartiles as well as 95% confidence intervals (CI). Means of continuous variables were compared using the independent samples *t*-test (age, initial aneurysm size) or the Mann-Whitney-U test (median follow-up (FU)) as appropriate. For comparison of categorical variables such as sex, multiplicity or smoking history, Pearson chi-square test or Fisher's exact test was used. Univariable logistic regression analyses were performed for all collected variables to calculate odds ratios and their 95% CI. Multivariable logistic regression was performed using the variables with a *p*-value of < 0.05 in the univariable analysis as well as smoking and hypertension (as these are established risk factors suggested in the literature for the formation and growth of intracranial aneurysms).

Survival analyses were conducted in the regular interval subgroup described in chapter 2.2.4. by plotting Kaplan Meier curves, calculating log rank tests to check for differences in survival curves as well as running univariable and multivariable Cox proportional hazard models to calculate hazard ratios and their 95% CI. For survival analyses, the first occurrence of growth was defined as event, while the end of follow-up in non-growing aneurysms was regarded as censored data.

Missing data was imputed using conditional multiple imputation method (“mice” package in RStudio®) for both logistic regression models and Cox proportional hazards model as appropriate.

An alpha-level of *p* < 0.05 was considered statistically significant. All statistical analyses were performed using RStudio® software (Posit®, Boston, USA) based on R version 4.3.1.

### 2.5 Ethical considerations

The analysis was conducted according to the International Declaration of Helsinki and was approved by the local ethics committee (number KEK, 2022-00430). Informed consent was obtained from all patients for retrospective collection, analysis and publication of anonymized patient data.

## 3 Results

### 3.1 Risk factor analysis

Patient- and aneurysm-related characteristics were calculated in a per-patient and a per-aneurysm based analysis.

#### 3.1.1 Per-patient analysis

588 patients met the inclusion criteria and were considered eligible for the analysis. Out of these, 404 (69%) were women and 184 (31%) were men. 358 patients had one aneurysm, and 230 patients had multiple aneurysms.

Table 1 displays the demographic variables for all patients as well as for the growing and the stable cohort individually.

**Table 1:** Per-patient analysis with baseline characteristics of n = 588 patients as well as the growing cohort (n = 103) and the stable cohort (n = 485). Means are presented with their corresponding standard deviation (mean ± SD), counts with their relative frequency (%) and odds ratios (OR) with their corresponding 95% confidence interval (CI). The first *p* value is calculated by descriptive statistics, the corresponding statistical test is represented at the bottom of the table. NF = neurofibromatosis; n/a = not available.

variable	total n (%)	growing n (%)	stable n (%)	*p* value	univariate analysis		multivariate analysis	
					OR (95% CI)	*p* value	OR (95% CI)	*p* value
no. of patients	588	103 (18)	485 (82)					
no. of aneurysms	858	112 (13)	746 (87)					

mean age ± SD (years)	58.1 ± 13.6	59.9 ± 12.5	57.7 ± 13.8	0.14^a^	1.01 (0.99 - 1.03)	0.14	n/a	n/a

sex								
men	184 (31)	25 (24)	159 (33)	0.09^b^	0.66 (0.40 - 1.07)	0.09	n/a	n/a
women	404 (69)	78 (76)	326 (67)					
number of aneurysms								
1 aneurysm	358 (61)	61 (59)	297 (61)	0.7^b^	1.09 (0.70 - 1.68)	0.70	n/a	n/a
> 1 aneurysm	230 (39)	42 (41)	188 (39)					
2	135	20	115					
3	56	13	43					
4	20	3	17					
5	11	3	8					
6	3	1	2					
7	0	0	0					
8	1	0	1					
9	3	2	1					
10	0	0	0					
11	0	0	0					
12	1	0	1					

smoking								
yes	337 (57)	64 (62)	273 (56)	0.20^b^	1.41 (0.89 - 2.26)	0.15	1.28 (0.80 - 2.06)	0.30
active	162	34	128					
former	161	28	133					
unknown	14	2	12					
no	218 (37)	31 (30)	187 (39)					
unknown	33 (6)	8 (8)	25 (5)					
hypertension								
yes	347 (59)	68 (66)	279 (58)	0.15^b^	1.53 (0.96 - 2.42)	0.07	1.45 (0.91 - 2.33)	0.12
no	225 (38)	31 (30)	194 (40)					
unknown	16 (3)	4 (4)	12 (2)					
aspirin intake								
yes	200 (34)	40 (39)	160 (33)	0.51^b^	1.28 (0.82 - 2.00)	0.28	n/a	n/a
no	361 (61)	59 (57)	302 (62)					
unknown	27 (5)	4 (4)	23 (5)					
family history of UIA or SAH (min. 2 positive)								
yes	7 (1)	3 (3)	4 (1)	0.20^c^	3.71 (0.79 - 17.43)	0.10	n/a	n/a
no	214 (36)	36 (35)	178 (37)					
unknown	367 (62)	64 (62)	303 (62)					
ADPKD								
yes	17 (3)	4 (4)	13 (3)	0.25^c^	1.41 (0.45 - 4.44)	0.55	n/a	n/a
no	543 (92)	97 (94)	446 (92)					
unknown	28 (5)	2 (2)	26 (5)					
connective tissue disease								
yes	9 (2)	1 (1)	8 (2)	0.15^c^	0.56 (0.07 - 4.52)	0.58	n/a	n/a
Ehlers-Danlos syndrome	0	0	0					
Marfan syndrome	0	0	0					
Fibromuscular dysplasia	5	1	4					
Loeys-Dietz syndrome	3	0	3					
NF Type 1	1	0	1					
no	545 (93)	100 (97)	445 (92)					
unknown	34 (6)	2 (2)	32 (7)					
history of SAH								
yes	76 (13)	12 (12)	64 (13)	0.67^b^	0.87 (0.45 - 1.68)	0.67	n/a	n/a
no	512 (87)	91 (88)	421 (87)					

a Independent samples *t*-test.

b Pearson chi square test.

c Fisher exact test (for small samples).

Univariable and multivariable logistic regression showed trends, but no statistically significant differences in the analyzed variables for growth between the growing and the stable cohort. Lower risk was observed in men compared to women (*p* = 0.09 in univariable logistic regression; OR 0.66 (95% CI 0.40 – 1.07)) and a higher risk in patients with hypertension (*p* = 0.07 in univariable and *p* = 0.12 in multivariable logistic regression; OR 1.45 (95% CI 0.91 – 2.33)) as well as in patients with a positive family history (*p* = 0.10 in univariable analysis; OR 3.71 (95% CI 0.79 – 17.43)).

#### 3.1.2 Per-aneurysm analysis

858 aneurysms were included in the per-aneurysm analysis. The mean patient age was 57.3 years (±12.4 years), and 619 aneurysms (72%) occurred in women. Median follow-up for the total cohort was 3.4 years (interquartile range IQR 1.3 – 6.6 years). 112 aneurysms (13%) showed growth (median follow-up 6.2 years, IQR 3.4 – 9.7 years), while 746 (87%) remained stable (median follow-up 3.2 years, IQR 1.2 – 6.1 years). 770 aneurysms (90%) were located in the anterior circulation and 88 aneurysms (10%) in the posterior circulation.

Table 2 summarizes the baseline characteristics for all aneurysms and displays the two subgroups of stable and growing UIA.

**Table 2:** Per-aneurysm analysis with baseline characteristics of n = 858 aneurysms as well as the growing cohort (n = 112) and the stable cohort (n = 746). Means are presented with their corresponding standard deviation (mean ± SD), counts with their relative frequency (%) and odds ratios (OR) with their corresponding 95% confidence interval (CI). The first *p* value is calculated by descriptive statistics, the corresponding statistical test is represented at the bottom of the table. NF = neurofibromatosis; n/a = not available.

variable	total n (%)	growing n (%)	stable n (%)	*p* value	univariate analysis		multivariate analysis	
					OR (95% CI)	*p* value	OR (95% CI)	*p* value
no. of patients	588	103 (18)	485 (82)					
no. of aneurysms	858	112 (13)	746 (87)					

mean age ± SD (years)	57.3 ± 12.4	58.7 ± 11.8	57.1 ± 12.4	0.21^a^	1.01 (0.99 - 1.03)	0.21	n/a	n/a

sex								
men	239 (28)	26 (23)	213 (29)	0.24^b^	0.76 (0.47 - 1.21)	0.24	n/a	n/a
women	619 (72)	86 (77)	533 (71)					
location of aneurysm								
anterior circulation	770 (90)	92 (82)	678 (91)	0.005^b^	2.17 (1.26 - 3.74)	0.005	2.01 (1.15 - 3.52)	0.01
ICA-cavernous (C4)	87	13	74					
ICA-intradural	224	12	212					
MCA	268	29	239					
ACA	186	38	148					
Other (ICA petrous etc.)	5	0	5					
posterior circulation	88 (10)	20 (18)	68 (9)					
VA	3	0	3					
PICA	7	2	5					
BA	25	6	19					
AICA	0	0	0					
SCA	11	2	9					
PCA	4	1	3					
PcomA	38	9	29					
smoking								
yes	530 (62)	71 (63)	459 (62)	0.20^b^	1.22 (0.78 - 1.91)	0.38	1.09 (0.68 - 1.74)	0.72
active	279	40	239					
former	229	29	200					
unknown	22	2	20					
no	285 (33)	32 (29)	253 (34)					
unknown	43 (5)	9 (8)	34 (5)					
hypertension								
yes	506 (59)	76 (68)	430 (58)	0.05^b^	1.66 (1.07 - 2.57)	0.02	1.60 (1.03 - 2.49)	0.04
no	332 (39)	32 (29)	300 (40)					
unknown	20 (2)	4 (4)	16 (2)					
aspirin intake								
yes	307 (36)	42 (38)	265 (36)	0.92^b^	1.09 (0.72 - 1.66)	0.68	n/a	n/a
no	521 (61)	66 (59)	455 (61)					
unknown	30 (3)	4 (4)	26 (3)					
family history of UIA or SAH (min. 2 positive)								
yes	32 (4)	6 (5)	26 (3)	0.51^b^	1.70 (0.66 - 4.40)	0.27	n/a	n/a
no	335 (39)	40 (36)	295 (40)					
unknown	491 (57)	66 (59)	425 (57)					
ADPKD								
yes	25 (3)	4 (4)	21 (3)	0.43^c^	1.25 (0.42 - 3.70)	0.69	n/a	n/a
no	799 (93)	106 (95)	693 (93)					
unknown	34 (4)	2 (2)	32 (4)					
connective tissue disease								
yes	10 (1)	1 (1)	9 (1)	0.08^c^	0.70 (0.09 - 5.59)	0.74	n/a	n/a
Ehlers-Danlos syndrome	0	0	0					
Marfan syndrome	0	0	0					
Fibromuscular dysplasia	6	1	5					
Loeys-Dietz syndrome	3	0	3					
NF Type 1	1	0	1					
no	795 (93)	109 (97)	686 (92)					
unknown	53 (6)	2 (2)	51 (7)					
history of SAH								
yes	134 (16)	17 (15)	117 (16)	0.89^b^	0.96 (0.55 - 1.67)	0.89	n/a	n/a
no	724 (84)	95 (85)	629 (84)					
mean aneurysm size at diagnosis ±SD (mm)	4.22 ± 2.89	5.17 ± 3.56	4.08 ± 2.75	0.0002^a^	1.10 (1.04 - 1.17)	0.0006	1.10 (1.04 - 1.16)	0.002

median FU time (lower quartile, upper quartile; years)	3.4 (1.3, 6.6)	6.2 (3.4, 9.7)	3.2 (1.2, 6.1)	<0.0001^d^	n/a	n/a	n/a	n/a

median time to growth (lower quartile, upper quartile; years)	n/a	3.6 (1.9, 8.0)	n/a					

a Independent samples *t*-test.

b Pearson chi square test.

c Fisher exact test (for small samples).

d Mann-Whitney-U test.

In the logistic regression analysis, the following variables were associated with UIA growth: UIAs located in posterior circulation (*p* = 0.005 in univariable and *p* = 0.01 in multivariable logistic regression; OR 2.01 (95% CI 1.15 – 3.52)), hypertension (*p* = 0.02 in univariable and 0.04 in multivariable analysis; OR 1.60 (95% 1.03 – 2.49)) as well as initial size at diagnosis (*p* = 0.0006 in univariable and *p* = 0.002 in multivariable analysis; OR 1.1 (95% CI 1.04 – 1.16) per mm).

##### 3.1.2.1 Survival analysis – cox proportional hazard regression models

As survival analyses are time-related and -dependent, they were performed with UIAs presenting with regular follow-up intervals of maximum 2 years between imaging studies only.

503 out of the total of 858 aneurysms met these criteria and were considered for the survival analyses.

For the same variables as in the analyses in Table 2, hazard ratios with the corresponding 95% CI as well as *p* values were calculated using univariable and multivariable Cox proportional hazard regression models (see Table 3). Aneurysm location (*p* = 0.001 in univariable and *p* = 0.002 in multivariable models; HR 3.9 (95% CI 1.74 – 8.75), age (*p* = 0.01 in univariable and *p* = 0.09 in multivariable models; HR 1.03 (95% CI 0.99 – 1.07) per increasing year of age) and initial size (*p* < 0.0001 in univariable and *p* < 0.0001 in multivariable analysis; HR 1.22 (95% CI 1.13 – 1.31) per mm increase in initial size) were associated with a shorter time to growth. Furthermore, there was a trend towards increased hazard in aneurysms of patients with hypertension (*p* = 0.07 in univariable and *p* = 0.12 in multivariable models; HR 1.94 (95% CI 0.86 – 4.51)). No statistically significant differences could be shown in terms of sex, smoking status, aspirin medication, family history, ADPKD, connective tissue disease as well as history of SAH.

**Table 3:** Cox proportional hazard regression model analysis of n = 503 aneurysms (i.e. the aneurysms with a regular follow-up of maximum 2 years interval between follow-up imaging studies) as well as the growing cohort (n = 37) and the stable cohort (n = 466). Means are presented with their corresponding standard deviation (mean ± SD), counts with their relative frequency (%) and hazard ratios (HR) with their corresponding 95% confidence interval (CI). NF = neurofibromatosis; n/a = not available.

variable	total n (%)	growing n (%)	stable n (%)	univariate analysis		multivariate analysis	
				HR (95% CI)	*p* value	HR (95% CI)	*p* value
no. of aneurysms	503	37 (7)	466 (93)				

mean age ± SD (years)	58.8 ± 11.1	63.3 ± 9.3	58.5 ± 11.1	1.05 (1.01 - 1.08)	0.01	1.03 (0.99 - 1.07)	0.09

sex							
men	135 (27)	10 (27)	125 (27)	0.93 (0.44 - 1.98)	0.84	n/a	n/a
women	368 (73)	27 (73)	341 (73)				
location of aneurysm							
anterior circulation	446 (89)	28 (76)	418 (90)	3.9 (1.78 - 8.54)	0.001	3.9 (1.74 - 8.75)	0.002
ICA-cavernous (C4)	37	2	35				
ICA-intradural	129	1	128				
MCA	164	8	156				
ACA	115	17	98				
Other (ICA petrous etc.)	1	0	1				
posterior circulation	57 (11)	9 (24)	48 (10)				
VA	1	0	1				
PICA	6	2	4				
BA	17	2	15				
AICA	0	0	0				
SCA	5	0	5				
PCA	3	0	3				
PcomA	25	5	20				
smoking							
yes	329 (65)	25 (68)	304 (65)	1.14 (0.55 - 2.34)	0.71	0.83 (0.39 - 1.76)	0.61
active	177	16	161				
former	139	7	132				
unknown	13	2	11				
no	155 (31)	12 (32)	143 (31)				
unknown	19 (4)	0 (0)	19 (4)				
hypertension							
yes	301 (60)	26 (70)	275 (59)	2.06 (0.94 - 4.51)	0.07	1.94 (0.86 - 4.51)	0.12
no	193 (38)	10 (27)	183 (39)				
unknown	9 (2)	1 (3)	8 (2)				
aspirin intake							
yes	184 (37)	15 (41)	169 (36)	1.12 (0.56 - 2.25)	0.75	n/a	n/a
no	303 (60)	21 (57)	282 (61)				
unknown	16 (3)	1 (3)	15 (3)				
family history of UIA or SAH (min. 2 positive)							
yes	25 (5)	2 (5)	23 (5)	0.89 (0.15 - 5.22)	0.89	n/a	n/a
no	205 (41)	13 (35)	192 (41)				
unknown	273 (54)	22 (59)	251 (54)				
ADPKD							
yes	9 (2)	0 (0)	9 (2)	0.0000001 (0 - ∞)	0.996	n/a	n/a
no	475 (94)	37 (100)	438 (94)				
unknown	19 (4)	0 (0)	19 (4)				
connective tissue disease							
yes	3 (1)	0 (0)	3 (1)	0.0000002 (0 - ∞)	0.997	n/a	n/a
Ehlers-Danlos syndrome	0	0	0				
Marfan syndrome	0	0	0				
Fibromuscular dysplasia	3	0	3				
Loeys-Dietz syndrome	0	0	0				
NF Type 1	0	0	0				
no	464 (92)	37 (100)	427 (92)				
unknown	36 (7)	0 (0)	36 (8)				
history of SAH							
yes	77 (15)	3 (78)	74 (16)	0.78 (0.23 - 2.66)	0.68	n/a	n/a
no	426 (85)	34 (92)	392 (84)				
mean aneurysm size at diagnosis ±SD (mm)	4.4 ± 3.1	6.3 ± 4.3	4.3 ± 3.0	1.20 (1.12 - 1.28)	<0.0001	1.22 (1.13 - 1.31)	<0.0001

##### 3.1.2.2 Survival analysis – Kaplan Meier curves

In addition to the hazard ratios of the Cox proportional hazard regression model, Kaplan Meier survival curves for all regularly followed-up aneurysms as well as for comparison of the aneurysm location (anterior vs. posterior circulation), hypertension (hypertension vs. no hypertension) and initial size (≤7 mm vs. > 7 mm) were plotted.

**Fig. 2:**
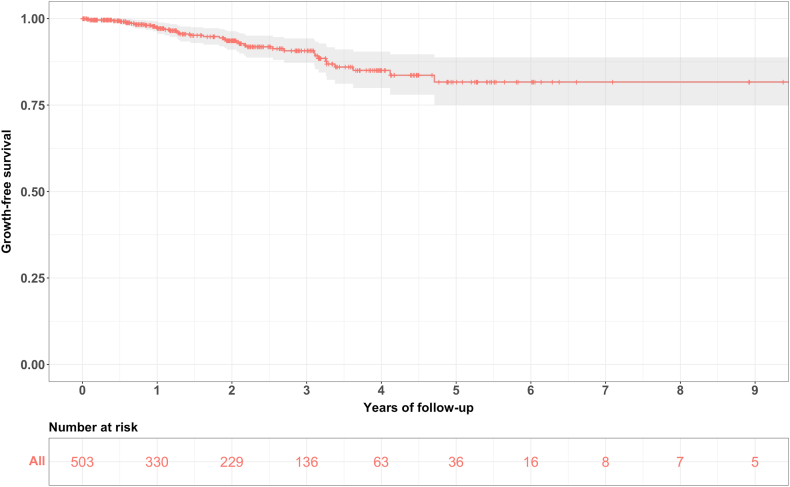
Kaplan Meier curve for all aneurysms with the corresponding 95% CI and the numbers at risk.

**Fig. 3:**
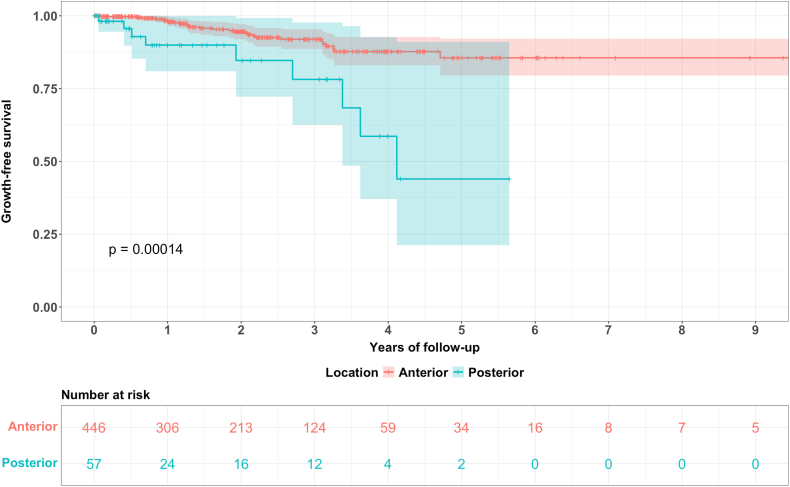
Kaplan Meier curves for aneurysms in the anterior vs. posterior circulation with the corresponding 95% CI and the numbers at risk, *p* = 0.00014 (log rank test).

**Fig. 4:**
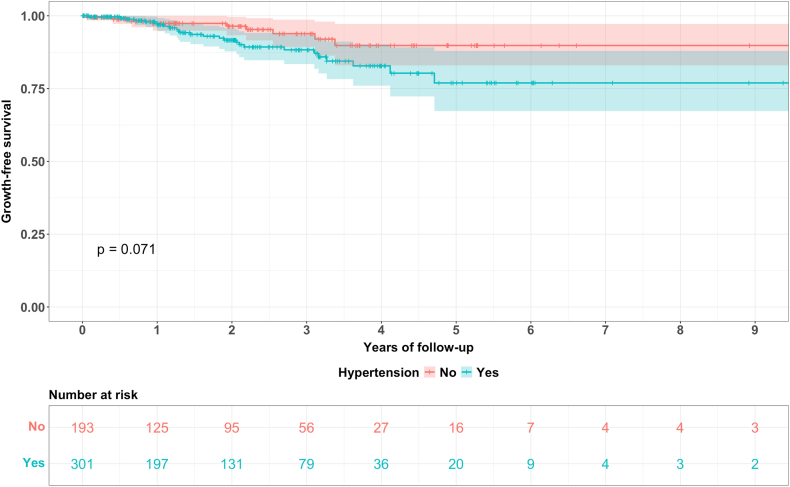
Kaplan Meier curves for aneurysms in patients with vs. without hypertension with the corresponding 95% CI and the numbers at risk, *p* = 0.071 (log rank test).

**Fig. 5:**
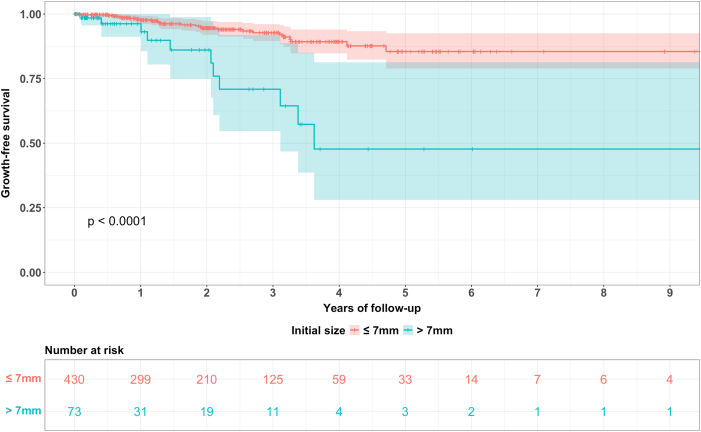
Kaplan Meier curves for aneurysms with an initial size of less than 7 mm vs. more than 7 mm with the corresponding 95% CI and the numbers at risk, *p* < 0.0001 (log rank test).

Fig. 2 shows the overall Kaplan Meier curve for all aneurysms, alongside the number of aneurysms at risk at different time points. Fig. 3, Fig. 4, Fig. 5 show the different survival curves in comparison of the 3 variables location, hypertension and initial size.

### 3.2 Incidence rate analysis

Table 4 shows an overview over different measures of incidence for different follow-up periods of time.

**Table 4:** Incidence counts and incidence rates over time. See chapter 2.2.5. for explanations on how the different columns were calculated.

time (years)	growing UIA count (n)	total UIA count (n)	UIA at risk (n)	Aneurysm-years at risk	Incidence rate (%; 95% CI)
0-2 years	21	274	503	679.30	3.1 (1.9 – 4.7)
2-4 years	13	166	229	268.05	4.8 (2.6 – 8.3)
4-6 years	2	47	63	71.75	2.8 (0.3 – 10.1)
6-12 years	1	16	16	26.95	3.7 (0.1 – 20.7)

Total	37	503		1046.06	3.5 (2.5 – 4.9)

In total, there were around 3.5 growing aneurysms per 100 aneurysm-years during the whole follow-up. This corresponded to an incidence proportion of 7.4% of all 503 regularly followed up aneurysms showing growth.

### 3.3 Imaging modalities in growth detection

As follow-up patterns were not standardized, we analyzed the frequency of different imaging modalities for aneurysm size measurement and growth assessments. Table 5 shows both the absolute number as well as a listing of the imaging modality combinations that detected growth (i.e. last imaging modality before growth and first imaging modality detecting growth). For the 112 growing aneurysms (compare Table 2), 163 events of growth were detected, as some aneurysms grew multiple times before undergoing treatment. In 121 out of 163 cases (74%), growth was detected in the same modality.

**Table 5:** Imaging modalities in total and imaging before vs. after growth occurrence. CTA = computed tomography angiography; MRA = magnetic resonance angiography; DSA = digital subtraction angiography.

variable	number n (%)
imaging modalities in total	4289
CTA	398 (9)
MRA	3047 (71)
DSA	844 (20)

imaging modalities in growth detection	163
CTA → CTA	7 (4)
MRA → MRA	109 (67)
DSA → DSA	5 (3)
CTA → MRA	15 (9)
CTA → DSA	0 (0)
MRA → CTA	7 (4)
MRA → DSA	3 (2)
DSA → CTA	4 (2)
DSA → MRA	13 (8)

## 4 Discussion

Different patient-und aneurysm-specific risk factors for both rupture and growth have so far been discussed in previous studies. Due to case selection, limited numbers or irregular follow-up patterns, results are inconsistent throughout the literature. With the present study, we add our data and results from a large neurosurgical and neurointerventional care centre in Switzerland to the risk factor research.

In the per-aneurysm logistic regression analysis, we found a location in the posterior circulation, the presence of arterial hypertension as well as the initial size at diagnosis to be associated with UIA growth. These results are consistent with the literature, demonstrating posterior location (Backes et al., 2016; Bor et al., 2015; Chien et al., 2013; Giordan et al., 2018; Lee et al., 2022), arterial hypertension (Jin et al., 2019; Jeon et al., 2014; Weng et al., 2020; Zanaty et al., 2020) and aneurysm size (Villablanca et al., 2013; Brinjikji et al., 2016; Backes et al., 2016; Jin et al., 2019; Bor et al., 2015; Giordan et al., 2018; Jeon et al., 2014; Backes et al., 2017; Backes et al., 2015; Björkman et al., 2019; Brinjikji et al., 2018; Chien et al., 2020; Igase et al., 2013; Serrone et al., 2016; Sonobe et al., 2010) as risk factors. With a OR of 1.60 (95% CI 1.03 – 2.49) for arterial hypertension and an OR of 1.10 (95% CI 1.04 – 1.16) for aneurysm size, we found comparable results to the pooled results from the systematic review of Jin et al. (arterial hypertension OR 1.18, 95% CI 1.05 – 1.32 (n = 374 aneurysms) and aneurysm size OR 1.34, 95% CI 1.15 – 1.56 (n = 539)) (Jin et al., 2019). Zanaty et al. for example presented larger effect size with an OR of 14.38 (95% CI 3.83 – 53.94) for arterial hypertension, but with a smaller population (Zanaty et al., 2020). For posterior location, we calculated an OR of 2.01 (95% 1.15 – 3.52), which resembles the results from Giordan et al. (OR 2.73, 95% 1.37 – 5. 44 (n = 385)) (Giordan et al., 2018).

Despite multiple studies showing an association of smoking with aneurysmal growth (Villablanca et al., 2013; Backes et al., 2016; Jin et al., 2019; Bor et al., 2015; Giordan et al., 2018; Brinjikji et al., 2018; Igase et al., 2013; Sonobe et al., 2010; Juvela, 2019), our results showed no clear correlation of smoking with the occurrence of growth (*p* = 0.38 in univariate and *p* = 0.72 in multivariate regression analysis). This may be due to incomplete data on smoking behaviour, as data was collected from medical reports which often were incomplete, missing the amount of pack years or the timing of smoking cessation.

Other individual studies have also found other factors such as age (Brinjikji et al., 2016; Backes et al., 2015; Kubo et al., 2014), female sex (Brinjikji et al., 2016; Jin et al., 2019; Sonobe et al., 2010; Juvela, 2019; Kubo et al., 2014; Inoue et al., 2012), ADPKD or other connective tissue diseases (Zanaty et al., 2020) to be associated with an increased risk of growth. In our study, these factors were not associated with growth.

In our survival analysis, posterior location and larger aneurysm size both were associated with a shorter time to growth which is in line with the literature (Bor et al., 2015; Jeon et al., 2014; Backes et al., 2017; Backes et al., 2015; Björkman et al., 2019; Sonobe et al., 2010). Putting our results of effect sizes into relation to the literature, we found similar results for posterior location with a HR of 3.9 (95% CI 1.74 – 8.75) compared to Bor et al. in their univariate analysis (HR 2.1, 95% CI 1.1 – 4.1 (n = 468)) (Bor et al., 2015). Comparing our aneurysm size HR of 1.22 (95% CI 1.13 – 1.31), different publications demonstrated comparable results as well, such as Jin et al. (HR 1.55, 95% 1.10 – 2.17 (n = 2381)), Bor et al. (HR 1.1, 95% CI 1.0 – 1.2 (n = 468)) or Björkman et al. (HR 1.35, 95% CI 1.19 – 1.52 (n = 350)) (Jin et al., 2019; Bor et al., 2015; Björkman et al., 2019). Due to variable FU patterns and our restriction to aneurysms followed up by a regular interval of maximum 2 years, the validity and power of our survival analysis results is limited.

Our study shows that aneurysms can grow or demonstrate instability after a significant time of seeming stability which may justify long-term follow-up strategies.

The overall incidence rate of around 3.5 growing aneurysms per 100 aneurysm-years over a FU period of around 10 years corresponds to rates described by Brinjikji et al. in their systematic review and meta-analysis (Brinjikji et al., 2016).

Larger prospective and long-term observational studies are needed to address the question whether certain patients or aneurysms do not have to be followed up regularly or even any more after a certain time.

One of the strengths of our study was the large number of patients and aneurysms compared to most studies that have been published so far, with over 100 growing and nearly 750 stable aneurysms as a control group. Even in the regular interval subgroup with around 500 aneurysms, our study remains one of the largest retrospective studies on this topic so far. Furthermore, as all patients diagnosed with unruptured intracranial aneurysms and referred to our institution are discussed on a weekly neurovascular board, the study population extracted from the board protocols reflects a realistic distribution of means and relative frequencies in terms of age, sex, aneurysm location etc. Unlike previous studies, we performed both logistic regression and Cox regression including survival analyses with a broad set of patient- and aneurysm-related variables.

An important limitation is the retrospective nature of our study. Firstly, this results in partially limited data availability and missing data. To account for that, multiple imputation methods were performed to impute missing data in logistic regression and Cox proportional hazard regression models. Secondly, the retrospective nature displayed many different follow-up patterns, intervals and total follow-up times ranging from a couple of days to > 20 years. Many patients and aneurysms were only followed up for a relatively short period of time due to dropouts or diagnosis at the end of the screening period. The different follow-up intervals are partly due to a relatively late introduction of standardized protocols regarding the interval and modality of FU imaging studies in stable UIAs in our institution during the second half of the screened board protocol timespan. To account for distortion of the mean time to growth by large follow-up intervals when actually growth might have occurred much earlier during that interval, we ran our survival analyses on a subgroup of aneurysms with a maximum interval of 2 years between FU imaging. The median follow-up time of the growing aneurysm cohort was significantly longer compared to the stable cohort (median 6.2 years, IQR 3.4 – 9.7 years vs. 3.2 years, IQR 1.2 – 6.1 years, *p* < 0.0001). This presumably biases our results to a certain degree but reflects common practice. As Nasiri et al., Skodvin et al. as well as Fargen et al. showed in their surveys, FU strategies vary in relation to modality and interval between large institutions across Europe and the United States as well, underlining the lack of guidelines (Fargen et al., 2018; Nasiri et al., 2024; Skodvin et al., 2021). Standardized, prospective observational studies with defined and standardized FU time intervals are needed to account for this problem, especially if long-term risks want to be addressed adequately.

Most analyses on the exact timing of growth are to a certain degree arbitrary, because most aneurysms are diagnosed incidentally and have been present for a long time already before the time of diagnosis.

Another limitation is the definition of growth in our study. Unlike most other studies on similar topics, growth was not defined as an increase in size of ≥ 0.5 mm or ≥ 1.0 mm in any dimension but rather a clear statement of growth or change in morphology in radiological reports and/or board protocols. This may compromise the applicability of our results because of a rather subjective than objective categorization in cases with a small increase in size. Furthermore, we based our categorization on radiological reports. This fact inherits the risk of interobserver variabilities between the different reporting radiologists. However, a strictly size-based definition of growth (e.g. increase in size ≥ 1 mm) has some disadvantages itself as well. Interobserver variability is relevant in size-based growth definitions as well. For aneurysm size changes in the range of the threshold, different readers will measure that size change differently with some surpassing the threshold and others not. Furthermore, with the limit of imaging resolution, especially in MRI/MRA, a strictly size-based growth definition in the range of the limit of imaging resolution inherits the risk of false assignments to either cohort as well. Timmins et al. showed in their study that the smallest detectable change for 2-dimensional aneurysm measurement ranges around 1.5-2 mm, which is significantly higher than the commonly used 1 mm definition (Timmins et al., 2021). As around 75% of growth occurrences were detected in the same modality (see Table 5), an inter-modality growth assessment was used in a minority of cases only, but requiring even clearer visual changes to reliably detect growth.

Current and future developments of automated measurement of UIA sizes and volumes by artificial intelligence might soon offer the possibility to objectify measuring aneurysms and therefore minimize interobserver variabilities as well as subjective assessment of growth.

Our analysis confirms the necessity and importance of acknowledging multiple factors and parameters in the multidisciplinary decision-making process on preventive treatment of unruptured intracranial aneurysms. The occurrence of growth as one of the most important risk factors for subsequent aneurysmal rupture implies the need for reliable correlations and associations to know which aneurysms are at a high risk of growth and rupture.

## 5 Conclusion

Posterior circulation location, the presence of arterial hypertension as well as a larger initial aneurysm size at diagnosis are associated with a higher risk of aneurysmal growth during follow-up. Institutional long-term regular follow-up strategies may be justified by the occurrence of growth after years of stability. Further observational studies are needed to gain more insight into long-term natural history of unruptured intracranial aneurysms and build long term follow-up protocols.

## Funding

Not applicable.

## Conflict of interest

The authors declare that they have no known competing financial interests or personal relationships that could have appeared to influence the work reported in this paper.
